# Effects of relaxation on depression levels in women with high-risk pregnancies: a randomised clinical trial**^1^**

**DOI:** 10.1590/1518-8345.1249.2806

**Published:** 2016-09-09

**Authors:** Wanda Scherrer de Araújo, Walckiria Garcia Romero, Eliana Zandonade, Maria Helena Costa Amorim

**Affiliations:** 2 MSc, RN, Hospital Universitário Cassiano Antonio de Mores, Universidade Federal do Espírito Santo, Vitória, ES, Brazil.; 3 PhD, Adjunct Professor, Universidade Federal do Espírito Santo, Vitória, ES, Brazil.; 4 PhD, Associate Professor, Universidade Federal do Espírito Santo, Vitória, ES, Brazil.

**Keywords:** Relaxation, Depression, Pregnancy, High-Risk, Nursing Care

## Abstract

**Objective:**

to analyse the effects of relaxation as a nursing intervention on the depression levels of hospitalised women with high-risk pregnancies.

**Methods:**

a randomised clinical trial realised in a reference centre for high-risk pregnancies. The sample consisted of 50 women with high-risk pregnancies (25 in the control group and 25 in the intervention group). The Benson relaxation technique was applied to the intervention group for five days. Control variables were collected using a predesigned form, and the signs and symptoms of depression were evaluated using the Edinburgh Postnatal Depression Scale (EPDS). The Statistical Package for Social Sciences (SPSS), version 20.0, was used with a significance level of 5%. The Wilcoxon and paired t-tests were used to evaluate depression levels between two timepoints. Using categorical data, the McNemar test was used to analyse differences in depression severity before and after the intervention.

**Results:**

depression levels decreased in the intervention group five days after the relaxation technique was applied (4.5 ± 3, p<0.05) compared with the levels at the first timepoint (10.3±5.9).

**Conclusion:**

as a nursing intervention, relaxation was effective in decreasing the symptoms of depression in hospitalised women with high-risk pregnancies.

## Introduction

Pregnancy can bring both joy and excitement, but many women experience sadness and anxiety, as pregnancy and the postpartum period involve many physical, hormonal, psychological and social changes that can have a direct impact on mental health^1^.

Furthermore, women with high-risk pregnancies are vulnerable to emotional changes, as they experience feelings of guilt and/or inadequacy, which can lead to feelings of uneasiness regarding their lives and the lives of their children^2^.

Depression is currently the most common mental disorder during pregnancy and the postpartum period. Approximately one in five pregnant women experience depression, but most are not diagnosed with or adequately treated for depression^3^.

The literature shows that some studies have focused on postpartum depression, but depression during pregnancy is also an important public health issue and a significant risk factor for postpartum depression, indicating the need for interventions before delivery^4^.

Depression during pregnancy appears to be more prevalent during the third trimester, with an incidence of approximately 20% in developing countries and between 10% and 15% in developed countries. Depression levels are higher in high-risk pregnancies, and depression is also associated with the following risk factors: a prior history of depression, financial difficulties, unemployment, a low educational level, a lack of social support, substance abuse, stressful events and domestic violence^5^. Symptoms of gestational depression have been correlated with other factors, including maternal anxiety, life stress, a prior history of depression, a lack of social support, domestic violence, an unwanted pregnancy and relationship factors^6^. The magnitude of these psychological changes will depend on biological, familial, conjugal, social and cultural factors and on the pregnant woman's personality^7^.

Studies have shown that depression has significant adverse effects on maternal and foetal health. In a systematic review of studies published from 1999 to February 2008 on the perinatal consequences of depression during pregnancy on the mother and the child, gestational depression was found to be a risk factor for postpartum depression, preeclampsia and premature labour, especially in pregnant women from low socioeconomic classes^8^. The main effects of depression on the foetus were associated with low birth weight^9^ ^-^ ^10^.

An analysis of the use of relaxation techniques to relieve pain during oncological treatment through progressive muscle relaxation, guided images, biofeedback, hypnosis and meditation concluded that these techniques reduced pain perceptions, nausea, stress and insomnia and that they acted as a coadjuvant for medications^11^. Yoga has also been used as an intervention. Indeed, yoga has been shown to reduce blood pressure in patients with arterial hypertension, and it can potentially be used as a non-pharmacological resource to aid patients with hypertension^12^. A study of patients in the postpartum period used the Benson relaxation technique described in this study and found a significant increase in the intervention group's immunoglobulin A (IgA) levels^13^.

Given the scientific evidence that pregnancy can lead to symptoms of depression that affect maternal and foetal health and that women with high-risk pregnancies are more vulnerable to these changes, the study reported here is necessary to aid nurses and healthcare teams in developing care plans for these pregnant women. Accordingly, we sought to analyse the effects of relaxation on depression levels in hospitalised women with high-risk pregnancies.

## Methods

### Study design

This randomised clinical trial was performed in the maternity ward of the Cassiano Antonio Moraes University Hospital in the city of Vitória (Espírito Santo, Brazil), which is recognised as a reference centre for high-risk pregnancies. The data were collected in 2013.

The author randomly assigned the pregnant women to the control and intervention groups when they were admitted, and no previous contact had been made with these women.

### Population and sample

The sample consisted of fifty hospitalised women with high-risk pregnancies at any gestational age. All were 18 years old or older and had been hospitalised for more than 24 hours in the maternity ward of the hospital. There were 25 women in the control group and 25 women in the intervention group. The following exclusion criteria were applied: a hearing or speech disability, a mental disability or dementia that might interfere with the interview or the intervention, and a hospital stay of less than five days.

The sample size was calculated for both groups by assuming the significance level to be 5% and the power of the test to be 80%. The minimum difference in the depression levels that we wanted to detect was 4, and the standard deviation in both groups was 5. Thus, an adequate size was obtained, with 25 pregnant women in each group. These data were generated with the Statistical Package for the Social Sciences (SPSS), version 18.0, with a random list of controls and interventions according to the spontaneous demand of the pregnant women. These women were then assigned to groups in that order. To avoid the Hawthorne effect, i.e., to avoid contaminating the subjects in the control group with those in the intervention group, some steps were taken; for example, patients in different groups were not allowed to be hospitalised in the same ward.

### Data collection

Every day, the records relating to the women with high-risk pregnancies who were able to participate in the study were selected. The researcher performed a random draw, and, to participate in the study, all the women who agreed to participate received informed consent forms and were guaranteed confidentiality. The researcher performed the interview, which included a signed form with sociodemographic and clinical variables, and applied the instrument and the intervention.

To measure the signs and symptoms of depression, the Edinburgh Postnatal Depression Scale (EPDS) was used. In Brazil, the EPDS is known as a self-evaluation scale for postpartum depression, and it is suggested to be better at identifying depressed individuals^14^. The EPDS is a self-administered instrument composed of ten items that are answered on a scale ranging from 0-3 based on the presence and intensity of the symptom. The statements cover the psychological symptoms of a depressed mood, including feelings of sadness, guilt, self-deprecation, thoughts of suicide or death, physiological symptoms (e.g., insomnia or hypersomnia) and changes in behaviour (e.g., crying spells). The total possible score is 30, with a score of 12 or higher indicating depression. Santos, Martins and Pasquali translated and validated the EPDS for Brazil^15^. It does not establish a formal diagnosis of depression but allows the intensity of depressive symptoms to be identified in order to refer individuals for evaluation and, if necessary, for treatment. The EPDS can be used during pregnancy or during the postpartum period. The author used this scale with the control and intervention groups during the first visit and five days later. The intervention groups were trained to use the Benson relaxation technique^16^, which includes four essential elements: a peaceful environment, a mental device, a passive attitude and a comfortable position. The six steps are as follows: (1) Sit quietly in a comfortable position. (2) Close your eyes. (3) Deeply relax all your muscles, beginning at your feet and progressing up to your face. Keep them relaxed. (4) Breathe through your nose. Become aware of your breathing. As you breathe out, say the word "one" silently to yourself. Breathe easily and naturally. (5) Continue for 10 to 20 minutes. When you finish, sit quietly for several minutes, at first with your eyes closed and later with your eyes opened. Do not stand up for a few minutes. (6) Do not worry about whether you are successful in achieving a deep level of relaxation. Maintain a passive attitude and permit relaxation to occur at its own pace. When distracting thoughts occur, try to ignore them by not dwelling on them and return to repeating "one."

Based on a previous study that used the Benson technique, which was adapted for a rehabilitation programme for women with mastectomies in Vitória (Espírito Santo, Brazil), this technique was chosen for use in this study^17^.

In the adapted technique, the following instructions were included between steps 5 and 6: "Now try to take a trip in your mind to a place where you have been and would like to return to or a place where you have never been and would like to go someday". This technique was individually taught to each pregnant woman for five days, such that they could learn and perform it twice a day, once upon waking and once before going to sleep.

### Variables

The following variables were assessed: age, marital status, educational level, spiritual support, occupation, social support, trait and state anxiety, smoking, alcoholism, gynaecological/obstetric history (number of births, the type of labour during previous pregnancies, and complications during previous pregnancies) and the current pregnancy (whether the pregnancy was planned and how many hospital admissions had occurred during the current pregnancy). These variables were collected using a specifically designed form, and they were controlled during the intervention study to achieve homogeneous groups.

### Data analysis

The SPSS, version 20.0, was used, and a significance level of 5%, corresponding to p<0.05 (95% confidence interval), was adopted. The chi-square test was used to measure the correlation between the control variables and the groups studied; the Mann-Whitney and unpaired t-tests were used to evaluate the relationship between the groups; and the Wilcoxon and paired t-tests were used to evaluate the depression level between the two timepoints. The McNemar test was used to analyse the differences in depression severity before and after the intervention using categorical data.

### Ethical considerations

This study was approved by the Research Ethics Committee of the Health Sciences Centre at the Federal University of Espírito Santo (Universidade Federal do Espírito Santo - UFES) under No. 237,302. After approval of the Ethics Committee, the data were collected and the instrument and relaxation technique applied from April 2013 to June 2013. In addition, each participant was told about the study goals and data collection procedure before they signed the written informed consent form. They were assured that anonymity and confidentiality would be guaranteed, along with the right to withdraw from the study at any time without being penalised. No pressure or inducement of any kind was applied to encourage the women to participate in the research. After the study ended, the women who were assigned to the control group were allowed access to the intervention.

## Results

The main causes of hospitalisation were hypertension, diabetes and haemorrhagic syndromes. The differences between the control and intervention groups were not significant for age, marital status, educational level, religion, occupation, social support, smoking, alcoholism, gynaecological/obstetric history and current pregnancy, demonstrating homogeneity between the groups, except in the case of labour type (p<0.05).

The control and intervention groups consisted largely of women who were younger than 29 years old (48% and 64%, respectively). Most lived with their partner in a stable relationship (96% in the control group and 92% in the intervention group).

Regarding educational level, 56% of the intervention group and 72% of the control group had somewhere between incomplete primary education and incomplete secondary education. Most reported that they were homemakers (48% in the control group and 76% in the intervention group). The study was conducted in the maternity ward of a public institution that mainly attends to low-income patients, which may explain the predominance of homemakers with low educational levels.

Most of the women did not consume alcohol or smoke cigarettes at that time - 96% for the two variables in the intervention group and 84% for the two variables in the control group. In both groups, the most common religion was evangelical (68% in the control group and 76% in the intervention group).

The pregnancy was not planned for most of the women (76% in the intervention group and 56% in the control group). Seventy-two per cent of the intervention group and 57% of the control group had complications during their previous pregnancies, likely because the sample comprised women with high-risk pregnancies. Vaginal childbirths were most common (65% in the intervention group and 40% in the control group), and most women had six or more prenatal visits during their current pregnancies. The women had had multiple pregnancies, with an average of 3.4 pregnancies in the control group and an average of 3.3 pregnancies in the intervention group. The mean number of births was 2.1 for the control group and 1.7 for the intervention group, while the mean number of abortions was 0.6 in both groups.

**Figure 1:**
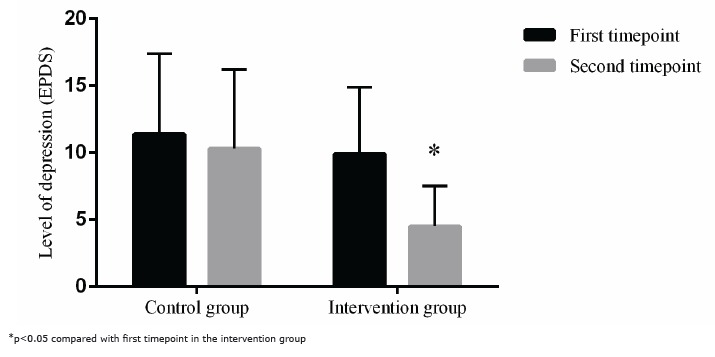
Comparison of the depression levels of women with high-risk pregnancies in the control and intervention groups at the first and second timepoints.

Overall, the gynaecological/obstetric data showed that the sample consisted of multiparous women with unplanned pregnancies who had experienced complications during their previous pregnancies. At the first timepoint, when admitted to the public maternity ward, the median level of depression measured by the EPDS was similar (p>0.05) in the control (11.4±6) and intervention (10.3±5.9) groups, indicating homogeneity between the groups. At the second timepoint, five days after hospital admission, there was no difference in the median EPDS score in the control group (9.9 ± 5), while the intervention group displayed a significant decrease (4.5 ± 3, p<0.05) compared with the median score at the first timepoint, as shown in Figure 1.

**Figure 2:**
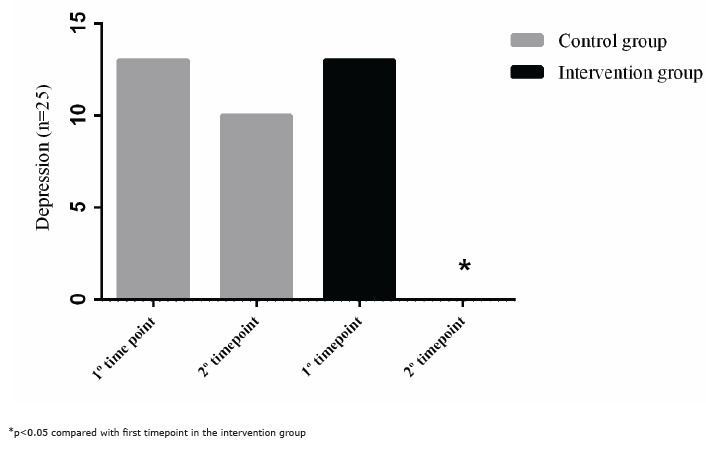
Distribution of depressed women with high-risk pregnancies at the first and second timepoints in the control and intervention groups.

Using a score of 12 as the cut-off for depression, the cases and controls were categorised into a non-depressed group (<12) and a depressed group (≥12). Figure 2 shows the results for the first and second timepoints for both the control and intervention groups. According to the McNemar test, there was no change in the depression level of the control group between the first (n=13) and second timepoints (n=10). However, the relaxation technique decreased (p<0.05) the symptoms of depression in hospitalised pregnant women between the first (n=13) and second timepoints (n=0).

## Discussion

This study has shown that relaxation as a nursing intervention was effective at significantly decreasing the levels of depression of hospitalised women with high-risk pregnancies.

Several authors have reported a decrease in stress factors due to relaxation^11^ ^-^ ^12^, but few studies have examined the effects of relaxation on women with high-risk pregnancies. Patients in the control group and intervention group had an EPDS score of greater than or equal to 12 at the first timepoint. This rate is high, possibly because this study collected data on hospitalised women with high-risk pregnancies and because the sample consisted mainly of multiparous women with unplanned pregnancies and complications in their previous pregnancies. In a study on quality of life, depression and anxiety in 120 primary care patients with high-risk pregnancies in the city of Campinas (São Paulo, Brazil), 32.5% exhibited symptoms of depression^18^. Another study analysed the incidence of depression in women with high-risk pregnancies and found that 50% of the women presented with symptoms of depression^19^. Another study analysed 712 pregnant women using the Primary Care Evaluation of Mental Disorders (PRIME-MD) instrument and found that 41.7% of the women probably had mental disorders^20^. These data are consistent with the results found in this study.

In a literature review investigating the prevalence of gestational depression in developing countries, including Brazil, a mean of 20% was found^5^, but none of the studies reviewed included women with high-risk pregnancies. In developed countries, the mean is slightly lower. In the United States, a cohort study found a 9% prevalence rate^21^; in an analysis of 2,430 prenatal patients in Switzerland, the rate was 13.7%^22^. The prevalence of depression tends to increase in women with high-risk pregnancies^5^; thus, diagnoses and early interventions after depressive symptoms are detected in these women is essential for reducing maternal and foetal risks.

Benson (1993) applied the relaxation technique to hypertensive patients and was able to reduce anxiety, blood pressure, nervousness and depression^16^. Additionally, anxiety decreases, and maternal-foetal attachment increases in primigravid women^23^.

As a limitation of this study, we highlight the small sample size due to our exclusion criteria, which excluded women with who were hospitalised for fewer than five days.

## Conclusion

Based on these results, relaxation as a nursing intervention is an important healthcare technology to help women with high-risk pregnancies reduce their depression symptoms. It is a simple practice that can be performed during hospitalisation, strengthening the nurse-patient bond and contributing to quality care. Therefore, it should be included in the daily nursing routine. Healthcare professionals, especially nurses, play an important role in recognising the diversity and intensity of the needs of women with high-risk pregnancies who seek integral care and the prevention of maternal and foetal complications.
